# Stress and Anxiety Levels Are Associated with Erythrocyte Fatty Acids Content in Young Women

**Published:** 2020-01

**Authors:** Samira Hashemi, Reza Amani, Bahman Cheraghian, Sorour Neamatpour

**Affiliations:** 1 Health Research Institute, Diabetes Research Centre, Department of Nutrition, Ahvaz Jundishapur University of Medical Sciences, Ahvaz, Iran.; 2 Department of Clinical Nutrition, School of Nutrition and Food Science, Food Security Research Center, Isfahan University of Medical Sciences, Isfahan, Iran.; 3 Department of Biostatistics and Epidemiology, Ahvaz Jundishapur University of Medical Sciences, Ahvaz, Iran.; 4 Department of Psychiatry, Golestan Medical Center, Ahvaz Jundishapur University of Medical Sciences, Ahvaz, Iran.

**Keywords:** *Anxiety*, *Dietary Pattern*, *Erythrocyte Fatty Acids*, *Psychological Disorders*, *Stress*

## Abstract

**Objective:** Recent studies have revealed that fatty acid profile can be associated with psychological disorders. However, evidence on stress and anxiety is scarce. The aim of this study was to investigate the relationship between stress and anxiety, defined as mood states, and erythrocyte fatty acid (FA) profile.

**Method** **:** This case-control study was conducted on 45 female students with degrees of stress and anxiety without depression disorder and 45 matched controls with no depression, stress, or anxiety. Self-administered questionnaires included a 28-item Food Frequency questionnaire and Depression Anxiety Stress Scales (DASS-21), which were used to measure dietary patterns and psychological disorders, respectively. Erythrocyte membrane fatty acids were analyzed using gas-liquid chromatography.

**Results: **Docosahexaenoic acid (DHA) was significantly lower in the case group (p = 0.008). Hydrogenated fats were associated with degrees of stress and anxiety (OR = 1.53, p = 0.019), while linoleate and DHA were inversely associated with stress and anxiety scores (OR = 0.37, p = 0.05; OR = 0.31, p = 0.014, respectively). Monounsaturated FAs (MUFAs) and total RBC trans FA were associated with increased risk of stress and anxiety (OR = 1.81, p < 0.001; OR = 3.38, p = 0.003, respectively).

**Conclusion: **Trans-fatty acids may be related to stress and anxiety scales but linoleate and DHA could decrease the risk. The effect of MUFAs may be regarded as a result of compensatory biological mechanisms.

Anxiety disorders are extremely common among the general population and defined as excessive fear and avoidance, often in response to specific objects or situations, and in absence of a real threat. An epidemiological study has indicated that the lifetime prevalence of any anxiety disorders is 28.8% in the US (1). It seems that the mental health pattern in Iran is almost similar to that of the Western countries (2). Medical students are exposed to psychological and physical stress due to the nature of their environment. Moreover, women are more vulnerable to most psychiatric disorders due to their higher stress levels when compared to the men (3). According to recent research, the prevalence of general anxiety in female medical students of Tehran University of Medical Sciences was 28.5% in 2010-2011(4). Stress response system (5), oxidative process (6), inflammation, brain plasticity and function (7, 8), known as biological processes, contribute to psychiatric disorders which can be affected by dietary pattern. The relationship between food and mood is potentially bidirectional. Although some authors suggest that the effects of food on mood are stronger and foods come first in the sequence of food-mood relationships (9). 

One of the most effective nutrients on mood and psychiatric disorders are fatty acids, and erythrocytes fatty acids content is a marker for long-term fatty acids intake (i.e. 120 days). 

Fatty acids as esters are present in phospholipids and their content can affect cell membrane fluidity (10). Vital structural and functional roles of eicosapentaenoic acid (EPA) and DHA in human brain have been recognised. They improve serotonergic neurotransmission, dendritic arborisation, synapse formation, prevent neuronal apoptosis, and modify ion channels (11, 12). Nevertheless, a vast number of studies indicate that essential fatty acids (EFAs) can be effective on psychiatric disorders (13, 14). However, other fatty acids are ignored, especially in relation to anxiety and stress. There is an inverse relationship between consumption of trans-fatty acids (TFAs) and depression/anxiety (15). 

Moreover, a possible relationship between fatty acids and stress and anxiety can indirectly be inferred from some studies. Data on a cross sectional study in 3 European countries revealed that in women, perceived stress was associated with more frequent consumption of sweet/fat foods and lower consumption of fruits/vegetables (16). In another study, as the first observational research on the role of DHA in anxiety disorders, it was concluded that women in the highest tertile of DHA intake experienced 50% lower anxiety disorders than the others (17).

Limited studies have investigated the relationship between fatty acids status and stress/anxiety, but none has studied the erythrocytes fatty acids content as the marker of long-term fatty acids intake. Therefore, this study aimed to assess the relationship between red blood cell (RBC) fatty acids profile and stress and anxiety scales in medical university female students. Consumption of the main food groups that could affect the fatty acid profile was also assessed.

## Materials and Methods

A case-control study was conducted on 45 female students with degrees of stress and anxiety without depression disorder and 45 controls with no depression, stress, or anxiety at Ahvaz Jundishapur University dormitories. The sample size was calculated as 82 persons, 41 students in each group. By estimating 10% dropout rate in the samples, the total sample size of 90 was obtained. To select the study groups, DASS-21 questionnaires were distributed among 500 volunteer students living in university dorms. In total, 358 students returned the questionnaires and were eligible to participate in the study. According to DASS questionnaire, different degrees of depression, stress, and anxiety were prevalent in 56.6%, 53.5%, and 33.7% of 358 students, respectively. Based on inclusion and exclusion criteria, groups were randomly selected from 156 students. According to the questionnaire, scores less than 14, 7, and 9 were defined as having no significant stress, anxiety, or depression, respectively. Students with scores above the related cutoffs had degrees of stress and anxiety. Also, students with depression (based on DASS questionnaire) were excluded from the study. Students were then selected by random sampling. A random sample of 45 students from those scoring above the cutoffs and a random sample of 45 from those below the scores were included as the cases and controls, respectively. The inclusion criteria were female students aged at least 18 years, living in university dormitories, and taking no medications or supplements which could have affected the blood variables level. Exclusion criteria were depression or any psychiatric disorders, except for anxiety and stress; also those on medications or dietary supplements were excluded. Written informed consent was obtained from all participants. The medical ethics committee at Jundishapur University approved the study protocol.

Variables

***Assessment of Dietary Intake***

For dietary assessment, students were asked to complete a validated food frequency questionnaire which included 28 main food items. The questionnaire was a modified version of FFQ that included food groups mainly consumed in students' dietary pattern (16). The introductory question, "How often do you eat the following foods?" was asked from all participants about the frequency of their usual consumption of each food group separately. The answers were rated on a 6-point scale: several times a day, daily, several times a week, 1–4 times a month, once in 2-3 months, and never. Content validity and reliability were evaluated through test – retest.

***Assessment of Psychological Scales***

Depression, anxiety, and stress status (DASS) questionnaire has been designed to measure 3 related but distinct negative affective states in nonclinical populations. This 21-item questionnaire contains 7 items for each of the 3 areas. Participants were asked to use a 4-pt severity/frequency scale to rate the extent to which they have experienced each state over the past week, where higher scores indicating a greater degree of mood disruption. The DASS takes approximately 5 minutes to complete, and the total score is determined by summing up the 3 subscale scores. Reliability of the 3 scales is considered excellent, with Cronbach's alpha at 0.95 for D, 0.90 for A, 0.93 for S, and 0.97 for the total score. Test-retest reliability is also excellent with 0.72 for D, 0.79 for A, and 0.81 for S. The DASS-D (depression) correlates with the Beck Depression Inventory (BDI-II; r = 0.74), and the standard clinical measure of depression (18). The DASS has adequate convergent and discriminant validity (CFI = 0.93)(19). Psychometric properties of the Persian version of DASS-21 are very similar to the results reported among a large nonclinical sample in the UK. The Cronbach's alpha was 0.94 for the total score of DASS-21. The Cronbach's alpha for Depression, Anxiety, and Stress scales was 0.85, 0.85, and 0.87, respectively (20). 

***General Characteristics***

The general questionnaire included the following information: age; weight; height; education level; marital status; habitual physical activity defined in 3 categories (3-5 days a week, 1-2 days a week, and no physical activity); and history of stress and/or anxiety disorders and psychological disease in participants and their families. In addition, medicines and dietary supplements used, having special diet in the last 6 months, and socioeconomic status defined in 3 categories based on the salaries (good, average, and poor) were recorded.

Individual's height and weight were measured while wearing light clothes with no shoes. Body mass index (BMI) was defined as weight (kg) divided by height squared (m2).

Blood Sample Collection and Analysis of Fatty Acids

Blood samples were taken after 12-hour night fasting. Whole blood samples were centrifuged at 2500 rpm for 10 minutes and the plasma and buffy coat were separated. Erythrocytes were washed with normal saline 3 times and stored at –80°C until further analysis. RBC fatty acids content was analyzed using gas-liquid chromatography (Buck Scientific 610, Norwalk, USA). The analyses for fatty acids were performed based on the classes of the major fatty acids: saturated fatty acids (SFAs: 14:0, 16:0, 18:0 and 20:0), trans-fatty acids (16:1t, 18:1t and 18:2t), total unsaturated fatty acids (UFAs: 16:1, 18:1, 18: 2, 18:3, 20:4, 20:5 and 22:6), mono unsaturated fatty acids (MUFAs: 16:1 and 18:1), omega-3 fatty acids (18:3, 20:5 and 22:6), and omega-6 fatty acids (18:2 and 20:4). 

***Statistical Analysis***

Statistical analyses were performed using SPSS version 20 (IBM SPSS, Chicago, IL). Comparison of dietary intake and concentrations of RBC fatty acids between the case and control groups were done using independent t test. Thereafter, regression analysis was applied to assess the relationship between dietary intakes and erythrocyte fatty acid content adjusted for confounding factors; then, odd ratios were calculated. BMI, age, economic status, and physical activity levels were included as the confounding factors in regression models. For all tests, -values less than 0.05 were considered statistically significant.

## Results

Basic characteristics of the study groups are presented in Table 1. Age, body weight, and BMI were not different between the groups. More than 80% of the cases had moderate and/or sever degrees of stress. Moderate and/or sever anxiety were found in 92% of the cases. 

Table 2 depicts the frequency of food groups consumption in the groups. None of the food subscales or food groups was associated with stress and anxiety scores.

Consumption of olive oil was marginally higher in controls (p < 0.063) (Table 2).

Table 3 presents the comparison of RBC fatty acids profile of the study groups. Compared to the controls, the sums of the total RBC trans and MUFAs were significantly higher in the stress and anxiety group (p = 0.002 and p < 0.001, respectively). The sum of polyunsaturated fatty acids (PUFAs) was marginally lower in the cases (p = 0.057). EPA concentrations were not different but those of DHA were significantly lower in the case group (p = 0.008). Linolenate (18:3) was significantly higher (p = 0.024) in the controls. Both trans forms of oleate and linolenate as well as oleate (p = 0.001) were significantly higher (p = 0.001 and p = 0.010, respectively) in the stress/anxiety group.

Table 4 shows odds ratios (95% confidence intervals) for RBC fatty acids profile and food items, pre- and post-adjustment. The crude ORs for linolenate (marginally), DHA, MUFA, and total trans were significant. Linolenate and DHA reduced the risk of stress and anxiety (OR = 0.037, p = 0.050 and OR = 0.306, p = 0.014, respectively). After adjustment, linoleate reduced the risk (OR = 0.46, p < 0.05). MUFAs and total trans increased the risk of these psychological disorders (OR = 1.812, p < 0.001 and OR = 3.382, p = 0.003, respectively). After adjustment, results for DHA and MUFAs remained significant. In addition to food items, hydrogenated fats increased the risk of stress and anxiety (OR = 1.53, p = 0.019). High fat dairies could also elevate the risk; however, the results showed a trend (OR = 1.63, p = 0.078).

**Table 1:** Basic Characteristics of the Two Study Groups

**Basic variables**		**Case (mean± SD)**	**Controls (mean± SD)**	***P***
Age		22.96(2.7)	23.58(4.0)	0.39
Weight		56.98(7.9)	56.44(7.5)	0.74
BMI		21.57(2.7)	21.71(2.5)	0.79
Students grade n (%)	BSc	29(46.4%)	25(55.6%)	0.58
	MSc	8(17.8%)	8(17.8%)	
	PhD	8(17.8%)	12(26.7%)	
Habitual physical activity	Yes	5(11.1%)	11(24.4%)	0.26
	No	30(66.7%)	26(57.8%)	
	Some times	10(22.2%)	8(17.8%)	
Socioeconomic status‡	Good	11(24.4%)	16(35.6%)	0.25
	Average	30(66.7%)	28(62.2%)	
	Poor	4(8.9%)	1(2.2%)	
Stress †	Mild	9(20%)	0	
	Moderate	17(37.8%)	0	
	Severe	17(37.8%)	0	
	Very severe	2(4.4%)	0	
Anxiety †	Mild	4(8.9%)	0	
	Moderate	22(48.9%)	0	
	Severe	9(20%)	0	
	Very severe	10(22.2%)	0	

‡ Based on salary

† Based on DASS questionnaire

**Table 2:** Consumption of the Main Food Groups by the Study Groups

**Food groups(servings)**	**Case (mean+SD)**	**Controls (mean+SD)**	***P***
Low fat dairy	2.97 ±1.196	2.80±0.99	0.445
High fat dairy	3.35±1.15	3.04±0.998	0.174
Fruits	2.80±0.842	2.53±0.786	0.124
Salads	3.26±0.986	3.088±0.763	0.342
Refined cereals	3.20±1.407	2.79±1.780	0.235
whole cereals(not refined)	2.08±1.018	2.16±0.974	0.729
Fast foods	4.31±0.668	4.33±0.707	0.879
Red meats	3.17±0.886	3.20±0.726	0.897
Fish	4.13±0.786	4.06±0.687	0.670
Eggs	3.88±0.647	3.68±0.90	0.229
Olive oil	4.86±1.337	5.33±0.977	0.063
Hydrogenated fats	4.73±1.388	4.25±1.705	0.155

**Table 3:** Comparison of RBC Fatty Acids Profile between the Study Groups

**Fatty acids profile** §	**Cases (mean±SD)**	**Controls (mean±SD)**	***P***
Myristat 14:00	1.161±0.596	1.293±0.390	0.217
Palmitate 16:00	37.646±3.198	38.128±6.316	0.649
Trans Palmitate 16:1t	0.495±0.109	0.514±0.200	0.577
Palmitoleate 16:1c	0.892±0.202	0.921±0.218	0.517
Stearate 18:00	18.128±1.578	17.400±3.652	0.223
Trans Oleate 18:1t	1.883±0.316	1.506±0.682	0.001
Oleate 18:1c	13.892±1.664	12.526±2.064	0.001
Trans linoleate 18:2t	0.851±0.103	0.780±0.146	0.010
Linoleate 18:2c	11.201±1.818	11.990±3.138	0.148
Linolenate 18:3c	0.336±0.215	0.436±0.195	0.024
CLA	0.368±0.233	0.475±0.364	0.102
Arashidate 20:00	0.598±0.294	0.658±0.176	0.247
Arashidonate 20:4c	11.288±2.303	1.725±3.694	0.503
EPA	0.423±0.750	0.494±0.118	0.529
DHA	0.832±0.507	1.148±0.594	0.008
SFA	57.534±3.258	57.480±5.512	0.954
MUFA	17.163±1.481	15.468±2.064	0.000
PUFA	25.301±3.237	27.051±5.153	0.057
Total trans	3.229±0.446	2.801±0.748	0.002
Total non-SFA	45.694±3.017	45.321±5.330	0.683

§ Fatty acid contents are expressed as a percentage of total fatty acids in erythrocytes. CLA: conjugated linoleic acid, EPA: eicosapentaenoic acid, DHA: docosahexaenoic acid, TFA: trans-fatty acids, SFA: saturated fatty acids, MUFA: monounsaturated fatty acids

**Table 4:** Odds Ratios (95% Confidence Intervals) for RBC Fatty Acids Profile and Food Items by the Study Groups

**Fatty acids**	**Crude OR(95%CI)**	***p***	**Adjusted OR(95%CI)**	***P*** *
linolenate	0.037(0.001-1.002)	0.050	0.042(0.002-1.176)	0.062
Linoleate	0.881(0.740-1.049)	0.155	0.463(0.226-0.949)	0.036
CLA	0.297(0.066-1.330)	0.112	0.544(0.030-9.879)	0.681
DHA	0.306(0.119-0.785)	0.014	0.159(0.032-0.775)	0.023
MUFA	1.812(1.326-2.475)	0	2.017(1.336-3.045)	0.001
PUFA	0.908(0.82-1.005)	0.061	0.997(0.710-1.399)	0.985
Total trans **Food items:** High fat dairy Hydrogenated fats	3.382(1.499-7.63) 0.485(0.196-1.199) 0.43(0.172-1.10)	0.003 0.117 0.82	2.038(1.437-3.241) 1.63(0.947-2.839) 1.53(1.072-2.192)	0.001 0.078 0.019

CLA: conjugated linoleic acid, DHA: docosahexaenoic acid, SFA: saturated fatty acids, MUFA: monounsaturated fatty acids

* variables were adjusted for lifestyle and dietary factors

## Discussion

Very few studies have investigated the relationship between RBC membrane fatty acids content and psychological disorders. This was the first study to evaluate the relationship between stress and anxiety status and RBC fatty acid profile. Previous studies have generally focused on omega-3 and/or omega-6 fatty acids and some types of psychological disorders, especially depression but they neglected stress/anxiety and other types of fatty acids, particularly trans-fatty acids. Results of a study on depression that measured RBC fatty acids found a direct association between total trans-fatty acids and MUFAs (with 18 or less carbon) concentrations in erythrocytes and depression, while PUFAs concentrations in the control group were higher (21). Apparently, TFAs may have an important role in psychological disorders. Results of Golomb et al study demonstrated a strong association between dietary TFAs and aggression (22). Moreover, in a cohort study by Sánchez-Villegas et al, a significant, direct, and dose-dependent association was found between dietary trans-fat intake and depression. Some other complications contributed to high TFAs consumption include inflammation, endothelial dysfunction, dyslipidemia, weight gain, and coronary heart disease. For MUFAs, this association was inverse (23). In a case control study by Pottala et al (2012), the authors concluded that RBC fatty acids are associated with depression. The concentrations of TFAs and MUFAs in RBCs were higher in the case group and, on the contrary, the PUFAs, particularly DHA, had an inverse association with depression. RBC fatty acids profile, when compared with plasma fatty acid levels, shows a longer time for intake of fatty acids. The life time of erythrocytes is approximately 120 days; therefore, it demonstrates fatty acids intake for about 4 months (24).

The causes of higher concentrations of erythrocyte MUFA, trans-fatty acids, and reduction in PUFAs may be multifactorial, which can be modulated by dietary factors, lifestyle, physical activity, and alteration in the activity of related enzymes such as elongase or desaturase (21). A possible hypothesis is that alterations in fatty acids may indicate a biological compensation for stress and anxiety. Increase in MUFAs may be due to its protective or adaptive role against cell membrane oxidation. When compared to PUFAs, MUFAs are more resistant to oxidative stress (21). Many studies have shown that oxidative stress increases stress and anxiety and other psychological disorders (25, 26). Oxidative stress occurs due to an imbalance between free radical oxygen species (ROS) and antioxidant defense. Because fat is the main content of the brain, its high oxygen consumption makes the brain vulnerable to oxidative impairment (27). Furthermore, proinflammatory cytokines can inhibit the expression of brain-derived neurotrophic factors (BDNFs) which play essential roles in axonal growth, neuronal survival, and plasticity of synapses (28). Some neuroprotection roles of this peptide are related to endothelial-produced BDNF. Therefore, fatty acid profile that promotes endothelial function for BDNF production, may have a protection role against psychological disorders, including stress and anxiety. Increased trans-fatty acids can be harmful for normal function of endothelial and reduce the production of BDNF (29, 30). Since fatty acids affect the membrane fluidity, especially that of neuron membrane which contains more fatty acids, they can affect neurological performance (31). 

Here, the relationship between dietary pattern and stress/anxiety status was also determined. None of the food subscales or food groups was associated with stress and anxiety scores after regression analyses for odd ratios. Adjusting food groups for age, BMI, economic level, physical activity, and marital status revealed that hydrogenated fats increased the risk of stress and anxiety. High fat dairies could also elevate the risk; however, the result was insignificant. 

The link between food and mood is a mutual relationship; in terms of the relationship between food and mood, the effects of food on mood is stronger (9). Studies on rats have indicated that stress can lead to activation of HPA axis and corticoid hormone release, which results in higher comfort foods intake. These foods are full of energy, carbohydrate, SFAs, and sodium (32). Studies on food and mood have not reached a consistent result, but they suggest that high fat and high carbohydrate foods can improve mood; nevertheless, their effect are short-termed (33). A study on college students indicated that higher intake of calorie, fatty acids, and sodium leads to negative mood 2 days later (9). 

Based on the results, consumption of olive oil was higher in the control group. Furthermore, intake of fat dairy was associated with higher stress and anxiety. Some parts of the results of the present study were consistent with other studies. In most studies, olive oil has shown a protective role for psychological disorders (23). A study comparing the traditional and Western diets in women showed that women with traditional diet consuming more vegetables, fruits, fish, and whole cereals significantly experienced lower stress and anxiety than women on Western diets who had more high fatty processed foods, refined cereals, and sweets (34). A cross sectional study revealed a positive association between saturated fatty acids and stress (35). Another research on middle age people indicated that full fat dairy has a positive association with stress, anxiety, depression, weak memory, and cognitive dysfunctions (36).

 The present study had some limitations. First, it did not evaluate desaturase and elongase enzymes activities/expression that can affect membrane fatty acids content. Second, stress and anxiety were evaluated using a questionnaire. Third, only female students were examined in this study because they are more vulnerable to psychological disorders; however, further studies should be conducted on both genders and with larger sample sizes. 

Food pattern and body fatty acids status may be related to stress and anxiety levels. TFAs are associated with increased risk of stress and anxiety; however, PUFAs, especially DHA, can help reduce this risk. Presumably, MUFAs, due to compensatory mechanisms, are increased because they have protective or adaptive roles against cell membrane oxidation. Further studies, preferably RCTs, are warranted to explore such relationships.

## Limitation

We were unable to carry out the study on larger population due to financial limitations and our findings need more evidence to confirm**. **Moreover, measuring specific markers such as BDNF could add a new area to this subject.

## Conclusion

Trans-fatty acids may be related to stress and anxiety levels while linoleate and DHA could decrease the risk. The effect of MUFAs could be regarded as a result of compensatory biological mechanisms.
